# Induction Chemotherapy in Technically Unresectable Locally Advanced Carcinoma of Maxillary Sinus

**DOI:** 10.1155/2014/487872

**Published:** 2014-05-11

**Authors:** Vanita Noronha, Vijay Maruti Patil, Amit Joshi, Muddu Vamshi Krishna, Sachin Dhumal, Shashikant Juvekar, P. Pai, Pankaj Chatturvedi, Devendra Arvind Chaukar, Jai Prakash Agarwal, Sarbani Ghosh, Vedang Murthy, Anil D'cruz, Kumar Prabhash

**Affiliations:** ^1^Department of Medical Oncology, Tata Memorial Hospital, Mumbai, India; ^2^Department of Radiodiagnosis, Tata Memorial Hospital, Mumbai, India; ^3^Department of Head & Neck Surgery, Tata Memorial Hospital, Mumbai, India

## Abstract

*Background*. Locally advanced carcinoma of maxillary sinus has been historically reported to have poor prognosis. We evaluated the role of NACT in improving the outcome in these patients. *Methods*. 41 patients with locally advanced technically unresectable (stage IVa) or unresectable maxillary carcinoma (stage IVb) were treated with induction chemotherapy between 2008 and 2011. The demographic profile, response and toxicity of chemotherapy, definitive treatment received, progression free survival (PFS), and overall survival (OS) were analyzed. Univariate and multivariate analysis were performed to determine factors associated with PFS and OS. *Results*. The chemotherapy included two drugs (platinum and taxane) in 34 patients (82.9%) and three drugs (platinum, taxane, and 5 FU) in 7 (17.1%). There was no complete response seen in any of the patients, stable disease in 18 (43.9%), partial response in 16 (39%), and progression in 7 (17.1%) patients. After induction, the treatment planned included surgery in 12 (29.3%), CT-RT in 24 (58.5%), radical RT in 1 (2.4%), palliative RT in 1 (2.4%), and palliative chemotherapy in 3 (7.3%) patients. Overall, the median PFS was 10.0 months. The OS at 24 months and 36 months was 41% and 35%, respectively. *Conclusion*. In unresectable maxillary carcinoma, induction chemotherapy has clinically significant benefit with acceptable toxicity.

## 1. Introduction 

Carcinoma of maxillary sinus is a rare entity [1]. It represents less than 1% of the tumours in the head and neck. Consequently, there is limited high-level evidence to define the ideal therapy in patients with maxillary tumours [1–6]. Epidemiologically, most of the cancers arising in this location are squamous cell carcinoma and are seen in advanced stage [1]. These tumours typically require extensive and morbid surgeries followed by adjuvant radiation. Though these procedures have been shown to improve outcomes, the long-term results are far from satisfactory [5–11].

Nearly 25–35% of patients have locally advanced unresectable disease [5, 11]. These patients have traditionally been treated with radical radiotherapy, with or without concurrent chemotherapy. The 5-year overall survival in this subgroup of patients is dismal, around 9–25% [7, 9, 12]. Considering such results, there is an urgent need for a new treatment paradigm for patients with maxillary carcinomas, especially in patents with unresectable disease or those who will require morbid resections.

Induction or neoadjuvant chemotherapy (NACT) prior to definitive local treatment has been used in carcinoma of maxillary sinus and response rates in the region of 60–70% have been reported. The survival with this approach was 77% at 2 years, as reported by Hanna et al. [3]. Since we had several patients with locally advanced and unresectable carcinoma we started using induction chemotherapy before definitive local treatment. The primary aim of NACT was to reduce the size of the tumour sufficiently to make them resectable. We present our results in locally advanced technically unresectable maxillary sinus tumours that were treated at our institute with NACT followed by reassessment for local treatment.

## 2. Material and Methods

**Figure 1:**
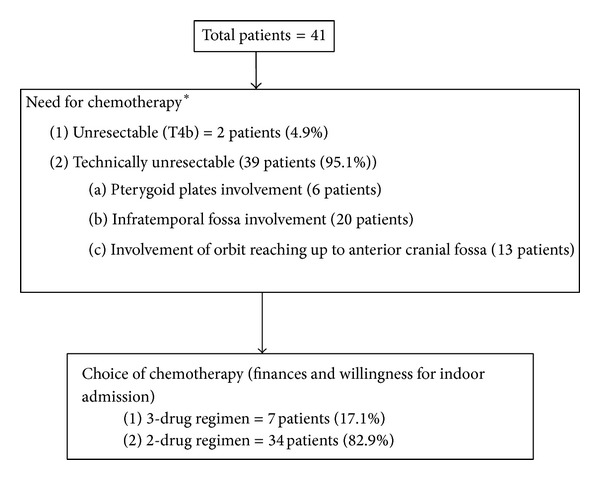
Flowchart of need for chemotherapy and choice of chemotherapy. *This decision about the reason for giving NACT was taken by multispecialty board.

All patients with squamous cell carcinoma of the maxillary sinus seen in the Head and Neck Medical Oncology Outpatient Department between January 2008 and December 2011 were evaluated. The patients treated with NACT were included in the present analysis. Data was collected on the demographics, baseline investigations, stage of the disease, indication of NACT, details of chemotherapy, chemotoxicity, response, post-NACT treatment details, failure pattern, and overall survival. The treatment for all patients at presentation was discussed in a multidisciplinary clinic. After clinical examination and radiological investigations, the tumour was considered either unresectable in advanced T stage (T4b) or technically unresectable when upfront surgery was likely to be associated with positive margins or result in an extremely morbid resection (Figure 1). These patients subsequently received 2 cycles of chemotherapy, either a 2-drug combination of platinum and taxane or a 3-drug combination of docetaxel, cisplatin, and 5 FU. The choice of chemotherapy depended upon the performance status, logistic issues, and financial status. Our personal experience had shown that the 3-drug regimen required a longer period of hospitalisation, prophylactic growth factors and was generally more expensive than the 2-drug regimens [13]. The platinum used depended on the serum creatinine clearance calculated in accordance with the modified Cockcroft Gault formula. If the creatinine clearance was above 60 mL/min, cisplatin was used. Carboplatin was the preferred agent when the creatinine clearance was below 60 mL/min. Standard doses of chemotherapy as previously established were used [13]. All the patients completed 2 cycles of chemotherapy and underwent axial imaging (either a contrast-enhanced computerised tomography or magnetic resonance imaging of the paranasal sinuses and cervical region) within 2–4 weeks. The response to therapy was quantified in terms of the RECIST criteria (response evaluation criteria in solid tumours). Further treatment was decided in the multidisciplinary clinic. In patients with stable disease or any response, patients either underwent definitive surgery followed by chemoradiation or definitive radiotherapy. Patients with progressive disease were treated with palliative intent. After the completion of therapy, patients were followed up with regular clinical examination and imaging whenever appropriate to document relapse and the pattern of relapse. All patients were followed up till death.

SPSS version 16 was used for statistical analysis. The demographic details, the response to induction chemotherapy, toxicity of induction, progression frees survival (PFS), and overall survival (OS) have been reported. Kaplan Meier analysis was used for estimation of time to event data.

PFS was calculated from date of first chemotherapy cycle to either date of progression or date of death whichever occurred earlier. The OS was calculated from date of first chemotherapy cycle to the date of death. Log-rank test was used for initial univariate analysis of factors affecting PFS and OS. The variables tested include age, gender, serum albumin, serum haemoglobin, body mass index, grade of tumour, response to NACT, drug regimen, dose intensity, and local treatment. Since multiple tests were used, Bonferroni correction was applied [14]. The *P* value was taken as significant if it was below 0.005.

## 3. Results 

Forty-one patients were included in the analysis. Two patients had T4b disease. The remaining patients with T4a tumours were considered technically unresectable due to involvement of pterygoid plates in 6 patients, infratemporal fossa involvement in 20, and orbital involvement up to the anterior cranial fossa in 13 patients.

The disease related details are presented in Table 1. All patients had squamous cell cancer. The grade of the tumour was well differentiated in 23 (56%), moderately differentiated in 10 (24.5%), and poorly differentiated in 8 (19.5%). The median age was 48 years (22–71 years). The sex ratio was 33 : 8. The median haemoglobin, albumin, and body mass index of these patients were 12.2 g/dL, 4 mg/dL, and 21.3 Kg/m^2^, respectively.

### 3.1. Chemotherapy Details

The median number of cycles of chemotherapy received was 2 (range 2-3). 34 patients (82.9%) were treated with 2-drug protocol. The chemotherapy given was docetaxel and cisplatin in 10 patients, docetaxel and carboplatin in 5, paclitaxel and cisplatin in 15, and paclitaxel and carboplatin in 4 patients. Seven patients (17.1%) were treated with a 3-drug regimen of docetaxel, cisplatin, and 5-fluorouracil. All patients completed at least 2 cycles of chemotherapy. The 20th quartile dose intensity was 85.4%.

The toxicity profile after chemotherapy was available for 38 patients and is depicted in Table 2. There were no deaths related to NACT. Serious adverse effects were febrile neutropenia in 8 patients (21.05%) and hyponatremia. All grades of hyponatremia were seen in 24 patients (63.16%) while grades 3-4 hyponatremia were seen in 11 patients (28.95%).

### 3.2. Response Evaluation Postchemotherapy

**Figure 2:**
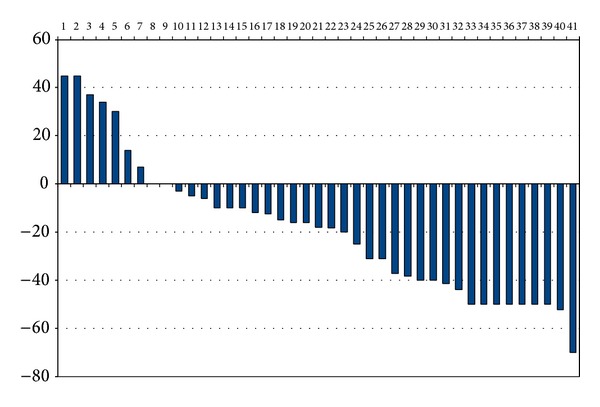
Waterfall plot of decrement in target size seen after 2 cycles of NACT among the patients. The *Y*-axis shows the change in target size. Positive figures indicate an increment in size while negative figures indicate a decrement in target size. The bars on *X*-axis represent each patient.

As per the RECIST criteria, complete response was seen in none, partial response in 16 (39%) patients, stable disease in 18 (43.9%), and progressive diseases in 7 patients (17.1%). The median decrement in size of the lesion was 20%. The waterfall plot of decrement in size of the lesion is given in Figure 2.

### 3.3. Post-NACT Definitive Treatment

After reassessment in the multidisciplinary clinic, the definitive treatment planned was surgery in 12 patients (29.3%), CT-RT in 24 (58.5%), radical RT in 1 (2.4%), palliative RT in 1 (2.4%), and palliative chemotherapy in 3 (7.3%) patients. However, 9 patients defaulted further treatment and 4 patients could not complete planned therapy due to logistical issues. Hence, the final therapy was surgery followed by postoperative chemoradiation in 8 patients, chemoradiation in 21, radical radiation in 1, palliative radiation in 1, palliative chemotherapy in 4, and no local treatment in 6 patients. Among the patients undergoing definitive surgery, 7 underwent total maxillectomy and one underwent suprastructure maxillectomy. The resection achieved was R0 in all patients. The margin status was close (2 mm or less of soft tissue margin) in 1 patient and was adequate in 7 patients. Pathological CR was achieved in 1 patient.

Among the patients receiving radiation, radical chemoradiation was given to 21 patients, radical radiation alone to 1 patient, adjuvant chemoradiation to 8, and palliative radiation to one patient. The median equivalent dose received by the tumour in these patients was 66 Gy (20–70 Gy). The median fraction time was 33 (10–33). The radiation techniques utilized were 3DCRT (3-dimensional conformal radiation technique) in 8 patients, IMRT (intensity modulated radiotherapy) in 8, and conventional planning in the remaining patients. Planned radiation course was completed by 27 patients.

### 3.4. Failure Pattern

**Figure 3:**
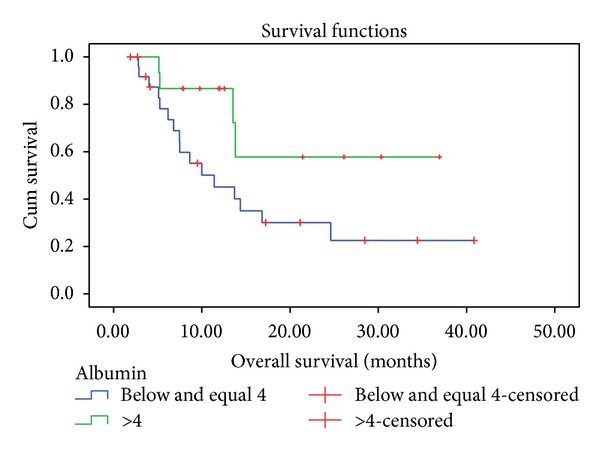
Survival graph showing effect of baseline albumin on overall survival. It was the only variable which affected the survival.

At a median followup of 36 months, 27 patients had failed. Local failure was the predominant type seen in 26 patients (96.3%). One patient had both local and distant failure. The local control rate was 34.1% at 3 years. The median PFS was 10 months. The OS at 24 months and 36 months was 41% and 35%, respectively. The variables evaluated included age, gender, serum albumin, haemoglobin, body mass index, grade of tumour, response to NACT, drug regimen, dose intensity, and local treatment. None of the variables affected the PFS and the only factor affecting OS was baseline serum albumin level (Figures 2 and 3).

## 4. Discussion

Carcinoma maxillary sinus is a challenging tumour to treat. The anatomical proximity to vital structures, locally advanced nature at presentation, and the biological aggressiveness have resulted in inferior outcomes compared to other head and neck subsites. There is a paucity of well-designed, large, and randomised studies specifically addressing maxillary cancers. As seen in Table 3, the studies are mostly retrospective case series which have low numbers, patients treated over decades, and both early and late stage carcinoma maxilla and include patients with malignancy of other paranasal sinuses [4–7, 9, 12, 15–22]. The available data suggests that in locally advanced tumours, surgery followed by radiation leads to better outcomes than radical radiation alone [5–8, 12]. There have been some studies on concurrent chemoradiation too that have shown benefit [4]. Overall, the reported survival in these studies has been between 34 and 40% at 5 years [2, 4, 23]. A previously published study from our centre by Qureshi et al. also reported a 3-year survival rate of 38% [9].

In the present study, we evaluated the role of induction chemotherapy followed by local therapy. We specifically wanted to assess the feasibility and effectiveness of surgery in patients following induction therapy whenever feasible. The primary reason for using NACT has been the discouraging results with radical radiation alone. In our study, all patients had squamous cell carcinoma, T4 disease, and around (2/3)rd of the patients (28 patients) had tumour invasion of the base of skull. All these have been documented to be poor risk factors in the literature [6, 19]. Despite this, our 3-year OS of 35% is comparable to our own published literature [9]. Significantly, in our previous study, 37.5% of patients had positive margins after surgery while in the present analysis, there was no margin positivity despite all the patients having upfront technically unresectable tumour (Table 4). Induction chemotherapy resulted in successful surgery in 12 patients out of 43 patients (27.9%) with technically unresectable patients.

Another factor which may have improved outcomes in our series is the use of advanced conformal radiotherapy techniques. Dirix et al. have previously reported on the use of 3D CRT (3-dimensional conformal radiotherapy) and IMRT (intensity modulated radiotherapy) at this site [18]. In their report, 2-year local control of 76% and OS of 89% were achieved. IMRT improved disease free survival from 60% to 72% compared to 3 DCRT (*P* = 0.002).

We used induction chemotherapy primarily consisting of 2 drugs with a taxane and platinum. The results with a similar regimen have been previously reported from Texas by Hanna et al. [3]. The response rate in our study is lower than the 67% reported in that study. The difference might be explained by the advanced stage of tumours in our study and the inclusion of other paranasal sinus malignancies in the study from Texas. The regimen was well tolerated, with an average relative dose intensity of more than 0.85 maintained by 80% of subjects and no treatment related mortality. The 2-drug protocol is easier to administer and needs fewer days of hospitalisation, which is an important consideration in resource-constrained countries. Though the numbers are small, the use of 3 drugs did not improve outcomes in terms of response rate, OS, and PFS.

The failure rate is significant and the majority occur in the first year. This pattern of early locoregional failure is consistent with previous literature [9, 12, 22]. By reducing the bulk of the tumour, induction chemotherapy facilitates surgery and also reduces the area of high risk CTV (critical tumour volume) in radiation planning.

## 5. Conclusion 

Induction chemotherapy may be effective in locally advanced technically unresectable maxillary cancers and leads to successful surgery in a significant proportion of patients. Our protocol of 2-drug regimen of taxane and platinum is effective and well tolerated. These findings are preliminary and require fsurther studies for confirmation.

## Figures and Tables

**Table 1:** Baseline tumor related parameters.

	Number (%)
T stage	
T3	2 (5%)
T4a	37 (90%)
T4b	2 (5%)
N stage	
N0	23 (56%)
N1	6 (14%)
N2	12 (30%)
N3	0
Stage grouping	
Stage IVA	39 (95%)
Stage IVB	2 (5%)
Grade of the tumour	
Grade 1	23 (56%)
Grade 2	10 (24.5%)
Grade 3	8 (19.5%)

**Table 2:** Toxicity details of induction chemotherapy (*n* = 38). The figures depicted are the number of patients.

	Grade 0	Grade 1	Grade 2	Grade 3	Grade 4
Anemia	7	21	10	1	0
Neutropenia	19	9	1	3	6
Thrombocytopenia	27	5	5	1	3
Vomiting	17	8	11	2	0
Loose motions	17	4	11	4	2
Mucositis	24	4	9	1	0
Fatigue	20	9	8	1	0
Hyponatremia	16	13	NA	7	4
Hypokalemia	28	7	NA	3	0
Febrile neutropenia	NA	NA	NA	3	5

**Table 3:** Selected results in carcinoma maxillary sinus.

Study	Number	Proportion of T4	Local control	OS
Paulino et al. [5]	48	50% (24)	Sx + RT (3 years)—65.2% RT (3 years)—22.7%	NR
Jansen et al.* [6]	73	59% (43)	Sx + RT (5 years)—65.0% RT (5 years)—47%	Sx+RT (5 years)—60.0% RT (5 years)—9%
Waldron et al. [15]	110	71% (78)	Five years—42%	Cause specific survival (5 years)—43%
Hayashi et al. [7]	62	48% (30)	CT-RT + Sx (5 years)—84% RT (5 years)—18.2%	CT-RT + Sx (5 years)—68.5% RT (5 years)—9.1%
Myers*et al. [16]	141	88% (124)	51% (median 336 days)	Five years disease specific survival—52%
Duthoy* et al. [17]	39	44% (17)	Two years—73% Cribriform plate invasion: 7 months	Two years—68%
Dirix et al. [18]	127		Five years: 53%	Five years 54%
Hoppe et al. [20]	85	52% (36)	Five years local progression free: 62%	Five years overall survival: 67%
Gabriele* et al. [12]	31		Sx + RT (5 years)—74.0% RT (5 years)—20%	Sx + RT (5 years)—72.0% RT (5 years)—25%
Hoppe et al. [20]	39	39 (100%)	Five years: 21%	Five years: 15%
Ramalingam et al. [22]	24	12 (50%)	Five years: 25%	Five years: 25%
Qureshi et al. [9]	73	36 (58.1%)	Three years: 54.8%	Three years: 38%
Jang et al. [4]	30	22 (73%)	Five years: 29%	Five years: 34%

*These studies which have included paranasal sinus patients rest are restricted to maxillary sinus carcinomas.

**Table 4:** Comparison of present series with previous published results from the same institute.

	Present series	Old series [9]
Number of patients	41	62
Time period	2008–2011	1994–1999
Stage IV	100.00%	58.50%
Induction chemotherapy	100.00%	0.00%
Radically treated	100.00%	100.00%
Margin positive rate	0.00%	37.50%
Three-year OS	35.00%	38.00%
